# Multi-drugs resistant acne rosacea in a child affected by Ataxia-Telangiectasia: successful treatment with Isotretinoin

**DOI:** 10.1186/s13052-015-0125-7

**Published:** 2015-03-28

**Authors:** Nicoletta Cantarutti, Alessia Claps, Giulia Angelino, Luciana Chessa, Francesco Callea, May El Hachem, Andrea Diociaiuti, Andrea Finocchi

**Affiliations:** Department of Systems Medicine, University of Rome “Tor Vergata”, Unit of Immunology and Infectious Disease, “Bambino Gesù” Childrens Hospital, Via Montpellier 1, 00133 Rome, Italy; Department of Clinical Molecular Medicine, University “La Sapienza”, Rome, Italy; Department of Pathology, Bambino Gesù Children Hospital, IRCCS, Rome, Italy; Department of Pediatrics, Unit of Dermatology, Bambino Gesù Children Hospital, IRCCS, Rome, Italy

**Keywords:** Ataxia-telangiectasia, Acne rosacea, Granulomas, Isotretinoin

## Abstract

Ataxia-Telangiectasia is a rare multisystem autosomal recessive disorder [OMIM 208900], caused by mutations in Ataxia-Telangiectasia Mutated gene. It is characterized by neurological, immunological and cutaneous involvement. Granulomas have been previously reported in Ataxia-Telangiectasia patients, even if acne rosacea has not been described.

We report a case of a young Ataxia-Telangiectasia patient with a severe immunological and neurological involvement, who developed granulomatous skin lesions diagnosed by skin biopsy as acne rosacea. Considering the severe clinical picture and the lack of improvement to multiple topic and systemic therapies, treatment with Isotretinoin was started and the skin lesions disappeared after five months. However the therapy was stopped due to drug-hepatotoxicity.

Systemic treatment with Isotretinoin should be carefully considered in patient with Ataxia-Telangiectasia for the treatment of multi-drug resistant acne rosacea, however its toxicity may limit long-term use and the risk/benefit ratio of the treatment should be evaluated.

## Background

Ataxia-Telangiectasia (A-T) [OMIM 208900] is a rare multisystem autosomal recessive disorder, caused by mutations in ATM (Ataxia-Telangiectasia Mutated) gene. It is characterized by cerebellar ataxia, oculocutaneous telangiectasias, oculomotor apraxia, variable immunodeficiency with recurrent infections, radiosensitivity and predisposition to malignancies. Cutaneous involvement usually includes telangiectasia, cafè-au-lait macules, hyper and hypopigmented macules and progeroid changes. Eczema and acanthosis nigricans may also be present, as well as vitiligo and granulomas [1].

Diagnosis is suggested by the clinical aspects, in according with elevated serum levels of alfa-fetoprotein and confirmed by detection of ATM protein expression by western blot analysis and identification of the gene mutations.

Cutaneous granulomatosis with unknown etiology, not related to infections, occur rarely in patients with primary immunodeficiencies (PIDs) [2]. These granulomas have been previously described in CVID, CGD, SCID, X- hypogammaglobulinemia, Wiskott-Aldrich syndrome and in A-T [2,3]. Particularly acne rosacea has not been described before in patients with A-T. We report a case of multi-drugs resistant acne rosacea in ataxia-telangiectasia, successful treated with Isotretinoin.

## Case report

A 15 years old girl, born from healthy Caucasian non-consanguineous parents, referred to our center at the age of 2 years with a history of failure to thrive and ataxic gate. The neurological picture was characterized by trunk and head ataxia, oculomotor apraxia, hypotonus, dysarthria, dysmetria and intention tremor. Physical examination revealed oculocutaneous telangiectasias, cafè-au-lait macules and striae rubrae. She suffered from recurrent infections, particularly by respiratory tracts infections and repeated episodes of fever of unknown origin. During follow up she developed chronic EBV infection that lead to arthritis EBV-related, requiring treatment with Rituximab at a weekly dose of 375 mg/m2 for 2 weeks. Immunological studies reveled a combined immunodeficiency, characterized by low level of serum IgA; non protective specific antibodies response against tetanus, after vaccination; T and B lymphopenia, associated with reduced naïve T subsets; low lymphocyte proliferation to PHA and skewed T Cell Receptor repertoire (Table 1). On the basis of the infections history and immunological assessment, intravenous immunoglobulin replacement therapy (IVIG) was started. Diagnosis of Ataxia-Telangiectasia (A-T) was confirmed by molecular analysis of ATM gene and by the evidence of ATM protein absence by western blot analysis.

**Table 1:** **Immunological data of the patients showing decrease level of IgA, T and B lymphopenia; reduced naïve T subsets (CD4RA,CD8RA); low lymphocyte proliferation to PHA; nonprotective specific antibodies response against tetanus, after vaccination; skewed T Cell Receptor repertoire**

**WBC/ml**		**9080**
Serum immunoglobulin mg/dl		
IgA		<5
IgM		187
IgG		1158
IgG1		906
IgG2		94.3
IgG3		10:01
IgG4		7.16
Lymphocyte phenotype (percentage and absolute number)		
Absolute count/ml		1260
CD3	33%	534.6
CD4	18%	291.6
CD4RA	1.6%	25.9
CD4RO	15.5%	251.1
CD8	11%	178.2
CD8RA	5.6%	90.7
CD8RO	5.2%	84.2
CD19	6%	97.2
CD16-56	50%	810
Response to mitogens (counts per min)		
PHA		19%
OKT3		48%
Response to vaccination		
Ab anti-Tet		0.1U/ml
Ab anti-PcP		50 mg/l
Ab anti-HiB		0.3 mg/l
Phenotype B		
CD22+CD27+IgD+IgM+		7.2%
CD22+CD27+IgD-IgM-		20.2%
CD22+CD27-IgD+IgM+		60.5%
CD21lowCD38NEG		28.7%
CD4+ and CD8+ TCR analysis (Spectratyping)		
CD4+ SKEWED		43%
CD8+ SKEWED		100%

**Figure 1:**
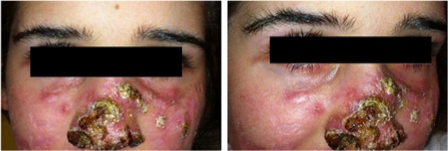
**Picture of the patient showing the presence of erythematous, papular-nodular lesions and telangiectasias on the face; ulceronecrotic lesions on the nasal pyramid with destructive effect on the wings of the nose.** Some scars are evident as result of the disease.

**Figure 2:**
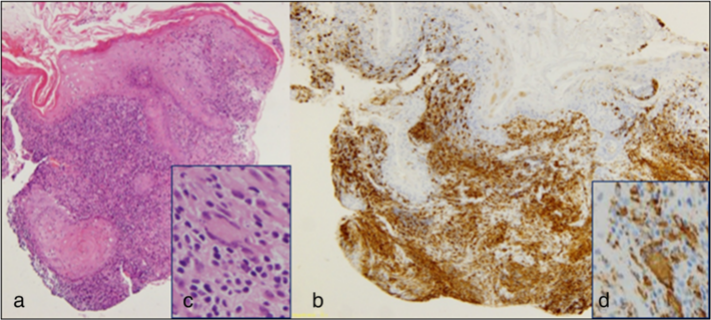
**Histological images of skin biopsy revealing a granulomatous inflammatory process.** The microphotograph shows a severely damaged epidermis due to a dense inflammatory process obliterating the entire dermis, mainly composed of histiocytes with an epithelioid appearance and lymphocytes arranged in a granulomatous pattern **(a)**. The immunostaining for CD68 shows positivity in the vast majotity of infiltrating cells **(b)**. A few multinucleated giant cell of Langhans type are present (**c**, insert) which are positive for CD 68 (**d**, insert). **a)** HE 10 x; **b)** Immunostaining 20 x; **c)** HE 40 x; **d)** Immunostaining 40 x.

At the age of 10 years the girl developed papules, pustules and granulomatous skin lesions with centrofacial distribution; a diagnosis of herpetic infection in immunocompromised patient has been suggested due to the presence of vesicular-pustular lesions with central umbilication. Treatment with acyclovir was started, in addition to systemic and local antibiotic therapy, but no improvement was observed. Because of the persistence and spreading of cutaneous lesions (Figure 1) and the negative result of DNA-HSV on the peripheral blood, a skin biopsy was performed and the histological study revealed a granulomatous inflammatory process rich in epithelioid histiocytes with a few multinucleated giant cells (Figure 2a-d). Stains for pathogens (Ziehl-Neelsen, PAS, Giemsa) were negative. The diagnosis of granulomatous acne rosacea was made. Rosacea fulminans was excluded because of the long lasting course characterized by a slow formation of new cutaneous lesions without draining sinuses. Moreover, on histology granulomas were striking while papillary dermis edema and deep dermal fibrosis were minimal. Therefore, the patient underwent oral and topical metronidazole treatment, but a significant worsening of the skin lesions with bacterial superinfection rapidly followed a slight improvement. Topical clindamycin was added to the ongoing therapy, while metronidazole was stopped due to detection of hypertransaminasemia at the blood tests. An assay with topical Pimecrolimus and systemic and topical Tetracycline was attempted, but, after a short period of improvement, a new worsening of the skin lesions occurred.

**Figure 3:**
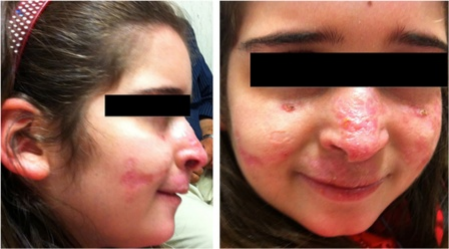
**Picture of the patient after five months of systemic treatment with Isotretinoin (0,5 mg/kg/die): the erythematous, papular-nodular and ulceronectrotic lesions disappeared with some residual pitted scars.**

**Figure 4:**
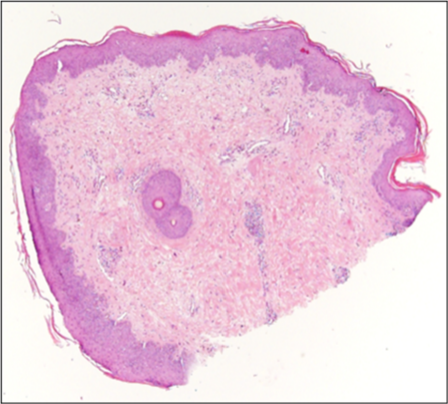
**Post-isotretinoin skin biopsy showing mild changes: slight hyperkeratosis, focal parakeratosis, acanthosis.** The dermis shows thickened collagen bundles, telangectasias and sparse or periadnexal lymphocytes. HE 4 x.

Considering the severe clinical picture and the lack of improvement to multiple therapies, systemic treatment with Isotretinoin (0,5 mg/kg/die) was started. The lesions showed mild improvement after four weeks and almost disappeared after five months (Figure 3), with residual pitted scars as documented histologically in a biopsy obtained from to same area (Figure 4). The treatment was continued for six months and then tapered, but a new worsening was observed. Therefore the therapy was resumed and stopped after five months due to drug-hepatotoxicity, with early relapse of skin lesions and progressive destruction of nose’s cartilage.

Cutaneous involvement is common in Ataxia-Telangectasia and usually it presents with cutaneous telangiectasia, skin atrophy, café-au-lait spots and premature graying. Cutaneous granulomas have occasionally been reported in patients with A-T [3-11], whereas acne rosacea has not been previously described in patients with A-T.

Acne rosacea is a chronic inflammatory disorder, characterized by centrofacial erythema, telangiectasias, papules, pustule and occasionally nodules or cysts on the forehead, cheek and nose. Rosacea can be classified in four different subtypes: erythemato-teleangiectatic, papulopustular, phymatous and ocular [12].

Cutaneous manifestations in immunocompromised patients have usually an atypical onset with chronic evolution and require more aggressive and prolonged therapy than non-immunocompromised one.

IVIG was reported to induce regression of the lesions in some patients with PIDs; other have described the development and persistence of cutaneous granulomas in patients on intravenous immunoglobulin replacement therapy [2,3]. Similarly, topic and systemic steroids in conjunction with antibiotics had no effects. Noteworthy, the cutaneous disease control in some PID’s patients has been obtained only after hematopoietic stem cells transplantation [2].

The conventional therapeutic regimens for acne rosacea, in non-immunocompromised patients, include topical and oral metronidazol, topical azelaid acid and oral doxycycline, in addition to adequate skin care [13]. Further interesting therapy option could be Isotretinoin, used off-label because of the lack of evidence-based data [14].

Isotretinoin is a synthetic retinoid derived from retinol, that is primary use orally for the treatment of severe, refractory, inflammatory acne. In literature is reported the efficacy of Isotretionin treatment, in patient affected by acne rosacea, at the dosage of 0.5 mg/kg/day. In particular its use is indicated for those patients who had experienced insufficient or transient effects with conventional topical and systemic therapies. The efficacy of Isotretinoin in rosacea is probably related to its anti-inflammatory, antioxidative, antiangiogenic, and antifibrotic properties, in addition to its ability to reduce the size of sebaceous glands [15].

Our patient is immunocompromised, she received multiple oral and topical treatments (steroids, mupirocin, metronidazole, tetracycline, clindamycin and pimecrolimus), without any improvement, whereas the lesions disappeared after five months of Isotretinoin, with residual pitted scars. In spite of its anti-inflammatory and immunomodulator properties, we did not observe an impairment of immune responses in our child, but on the other side a severe hepatotoxicity was detected and the treatment was stopped.

## Conclusion

Due to our experience, the choice of Isotretinoin should be carefully considered in patients with A-T for the treatment of acne rosacea resistant to conventional therapies, however its toxicity may limit long-term use and the risk/benefit ratio of the treatment should be evaluated.

## Consent

Written informed consent was obtained from the parents of the patient for publication of this Case report and any accompanying images. A copy of the written consent is available for review by the Editor-in-Chief of this journal.

## Abbreviations

A-T: Ataxia-Telangiectasia
ATM: Ataxia-Telangiectasia mutated
IVIG: Intravenous immunoglobulin replacement therapy
PIDs: Primary immunodeficiencies
